# DeepAVPred: A Deep Learning Framework Integrating RECM-DWT and BiGRU for Accurate Antiviral Peptide Prediction

**DOI:** 10.3390/ijms27209086

**Published:** 2026-10-13

**Authors:** Huma Fida, Hong-Qi Zhang, Basharat Ahmad, Hao Lin, Cai-Yi Ma, Cheng-Bing Huang, Zhi-Yu Fan

**Affiliations:** 1 School of Life Science and Technology and Center for Informational Biology, University of Electronic Science and Technology of China, Chengdu 610054, Chinacy_ma@abtu.edu.cn (C.-Y.M.); 2 School of Computer Science and Technology, Aba Teachers College, Aba 623002, China

**Keywords:** antiviral peptides, machine learning, deep learning, feature extraction, drug discovery

## Abstract

Viral infections continue to pose a significant global health challenge. Antiviral peptides (AVPs) have emerged as promising therapeutic agents owing to their high specificity, favorable safety profiles, and broad-spectrum antiviral activity. However, experimental AVP identification is time-consuming and costly, while existing computational predictors often have limitations in predictive accuracy and feature representation. Here, we propose DeepAVPred, a deep learning-based framework for AVP identification. DeepAVPred integrates complementary sequence descriptors to capture physicochemical properties and pairwise residue interactions. We present a novel feature representation, the Residue Energy Content Matrix augmented with a Discrete Wavelet Transform (RECM-DWT). RECM-DWT characterizes multi-scale patterns in residue-energy information and complements conventional sequence descriptors by providing wavelet-derived features across multiple resolution levels. The integrated features are then modeled using an attention-based Bidirectional Gated Recurrent Unit (BiGRU) network to learn contextual dependencies and informative sequence patterns. On the AVP_train_ dataset, DeepAVPred achieved strong performance in 10-fold cross-validation, with an accuracy of 0.990, a Matthews Correlation Coefficient (MCC) of 0.980, and an area under the receiver operating characteristic curve (AUC) of 0.993. On the independent test dataset, AVP_test_, DeepAVPred achieved an accuracy of 0.942, an MCC of 0.886, and an AUC of 0.949. These results demonstrate the predictive performance of DeepAVPred and support its potential as a computational framework for prioritizing candidate antiviral peptides for further experimental validation.

## 1. Introduction

Viral infectious diseases have posed persistent and serious threats to global public health throughout human history. From the influenza pandemic of the late 1960s and the long-standing burden of HIV/AIDS to the recent COVID-19 pandemic, viral outbreaks continue to emerge, resulting in profound medical, social, and economic consequences [1,2,3,4]. These events highlight the ongoing challenges associated with the rapid development, large-scale production, and timely distribution of effective antiviral drugs and vaccines. Viruses remain particularly difficult to control due to their high mutation rates, efficient transmission mechanisms, and ability to evade host immune responses [5]. Although approximately 90 antiviral drugs have been approved for clinical use, they target only a limited number of viral families, leaving a significant proportion of the more than 200 human-pathogenic viruses without effective treatments [6]. Moreover, emerging and re-emerging viral threats, such as Zika virus, Ebola virus, and drug-resistant viral strains, further emphasize the urgent need for novel and broadly effective antiviral strategies. Peptide-based therapeutics have attracted increasing attention as promising alternatives to conventional small-molecule antivirals due to their high specificity, favorable safety profiles, and relatively low production costs [7].

Peptides, typically consisting of 2–30 amino acids, play essential biological roles as signaling molecules, hormones, and antimicrobial agents. Among these, antiviral peptides (AVPs) represent a distinct and valuable class of therapeutic candidates capable of inhibiting viral infection through diverse mechanisms, including disruption of viral membranes, inhibition of viral entry, and interference with viral replication. Recent AVP-specific repositories contain over 2600 validated antiviral peptide entries, reflecting the growing interest in AVP research and development [5]. Despite their therapeutic potential, the discovery and characterization of AVPs remain challenging. Experimental identification methods are labor-intensive, costly, and time-consuming, making them impractical for large-scale peptide screening [8,9,10]. Consequently, machine learning (ML)- and deep learning (DL)-based computational approaches have become important strategies for predicting peptide bioactivity and distinguishing AVPs from non-AVPs [11,12].

AVPred [13], Chang et al.’s random forest-based method [14], AVP-IC50Pred [15], AVCpred [16], Meta-iAVP [17], AntiVPP 1.0 [18], FIRM-AVP [19], DeepAVP [20], ACVPred [21] and ENNAVIA [22] have been developed for AVP prediction. Although these methods have advanced AVP prediction, limitations remain in representing complex sequence patterns and extracting informative features from peptide sequences, motivating the development of improved feature representations and modeling approaches. These limitations highlight the need for improved feature representation and modeling approaches, which motivated the development of DeepAVPred. 

To address these limitations, we propose DeepAVPred, a deep learning-based framework for AVP identification. DeepAVPred is designed to integrate complementary feature representations and sequence modeling to improve AVP identification. It introduces a new representation, RECM-DWT, that captures both short-term and long-term changes in residue interactions, providing information that conventional descriptors may not capture. By combining RECM-DWT with an attention-based BiGRU architecture, DeepAVPred enhances the representation of peptide properties and improves predictive performance. DeepAVPred achieved an accuracy of 0.990 and an MCC of 0.980 on the AVP_train_ dataset and an accuracy of 0.942 and an MCC of 0.886 on the independent AVP_test_ dataset. For AVP_test_, the accuracies reported for AVPred [13], Chang et al.’s method [14], Meta-iAVP [17], DeepAVP [20], and ENNAVIA [22] ranged from 0.925 to 0.949, and their MCC values ranged from 0.850 to 0.900. DeepAVPred’s results fall within the upper part of these reported ranges, although differences in preprocessing and evaluation protocols limit direct cross-study comparisons. The performance of DeepAVPred was systematically evaluated and compared with several state-of-the-art machine learning and deep learning models, including Support Vector Machine (SVM) [23], Random Forest (RF), Light Gradient Boosting Machine (LightGBM), Multi-Layer Perceptron (MLP), Deep Forest, and conventional BiGRU architectures. Extensive experiments using cross-validation and independent test datasets demonstrate the competitive predictive performance of DeepAVPred across the evaluated datasets and metrics. Overall, this study presents an effective computational framework that combines advanced feature engineering with deep learning to facilitate high-confidence AVP identification. The proposed DeepAVPred model provides a scalable tool for peptide-based antiviral drug discovery and large-scale screening of therapeutic peptides.

## 2. Results

### 2.1. Ablation Analysis of CPSR Features on *AVP_train_* and *AVP_test_*

To evaluate the discriminative capability of CPSR features, multiple machine learning (ML) and deep learning (DL) models were trained and evaluated on the AVP_train_ and AVP_test_ datasets. The performance metrics obtained through 10-fold cross-validation and independent testing are summarized in Table 1. In 10-fold cross-validation on the AVP_train_ dataset, Deep BiGRU achieved the highest classification performance, attaining an accuracy of 0.939 and an MCC of 0.8795, suggesting that CPSR features provide useful discriminatory information for AVP classification. Traditional ML models such as Random Forest and LightGBM also demonstrated competitive performance, whereas SVM exhibited comparatively weaker results. When evaluated on the independent AVP_test_ dataset, Random Forest marginally outperformed the deep learning models, achieving an accuracy of 0.933 and an MCC of 0.8744.

### 2.2. Ablation Analysis of ExPseAAC Features on *AVP_train_* and *AVP_test_*

The second ablation experiment assessed the predictive effectiveness of ExPseAAC features across the same set of classifiers. As shown in Table 2, ExPseAAC features showed varying performance across the evaluated ML and DL models compared with CPSR. In 10-fold cross-validation on the AVP_train_ dataset, Deep BiGRU achieved an accuracy of 0.921 and an MCC of 0.845. Random Forest and LightGBM again showed competitive performance among the evaluated models. On the independent AVP_test_ dataset, LightGBM achieved the highest accuracy (0.900) and MCC (0.800), while Deep BiGRU followed closely.

### 2.3. Ablation Analysis of RECM Features on *AVP_train_* and *AVP_test_*

Residue Energy Content Matrix (RECM) features were employed to incorporate pairwise residue interaction energies into the prediction framework. The results summarized in Table 3 indicate improved performance with RECM features compared with CPSR and ExPseAAC features. In 10-fold cross-validation on the AVP_train_ dataset, Deep BiGRU achieved the highest accuracy (0.921) and MCC (0.843), indicating effective performance with the RECM feature representation. Traditional ML classifiers, particularly Random Forest and LightGBM, also showed competitive performance with RECM features, suggesting that residue interaction energies provide useful discriminative information. On the AVP_test_ dataset, Random Forest achieved the best performance, with an accuracy of 0.900 and an MCC of 0.807.

### 2.4. Ablation Analysis of RECM-DWT Features on *AVP_train_* and *AVP_test_*

To further enhance feature representation, a novel encoding method, RECM enhanced with a Discrete Wavelet Transform (RECM-DWT), was introduced. The comparative results in Table 4 show that RECM-DWT achieved higher predictive performance than the other evaluated feature encoding strategies. The overall performance of the proposed Deep BiGRU model was evaluated using the mean and SD of accuracy, sensitivity, specificity, MCC, and AUC values obtained from the 10-fold cross-validation on the AVP_train_ dataset: 0.990 ± 0.032, 0.987 ± 0.040, 0.993 ± 0.023, 0.980 ± 0.063, and 0.993 ± 0.021, respectively. However, a marked difference in model performance was observed during evaluation on AVP_test_. Deep BiGRU achieved an accuracy of 0.942 and an MCC of 0.886, while Random Forest achieved an accuracy of 0.916 and an MCC of 0.835. In contrast, LightGBM and MLP showed substantial performance deterioration. Most notably, LightGBM did not correctly identify any negative samples, resulting in a specificity of 0.000 and an MCC of 0.000. Further examination of the corresponding confusion counts showed that LightGBM produced TP = 60, FN = 0, TN = 0, and FP = 60, indicating that all 120 AVP_test_ samples were classified as positive. This behavior is consistent with its sensitivity of 1.000, specificity of 0.000, accuracy of 0.500, and MCC of 0.000. For MLP, the corresponding counts were TP = 57, FN = 3, TN = 16, and FP = 44, consistent with its high sensitivity (0.950) but low specificity (0.266) and MCC (0.296).

**Figure 1:**
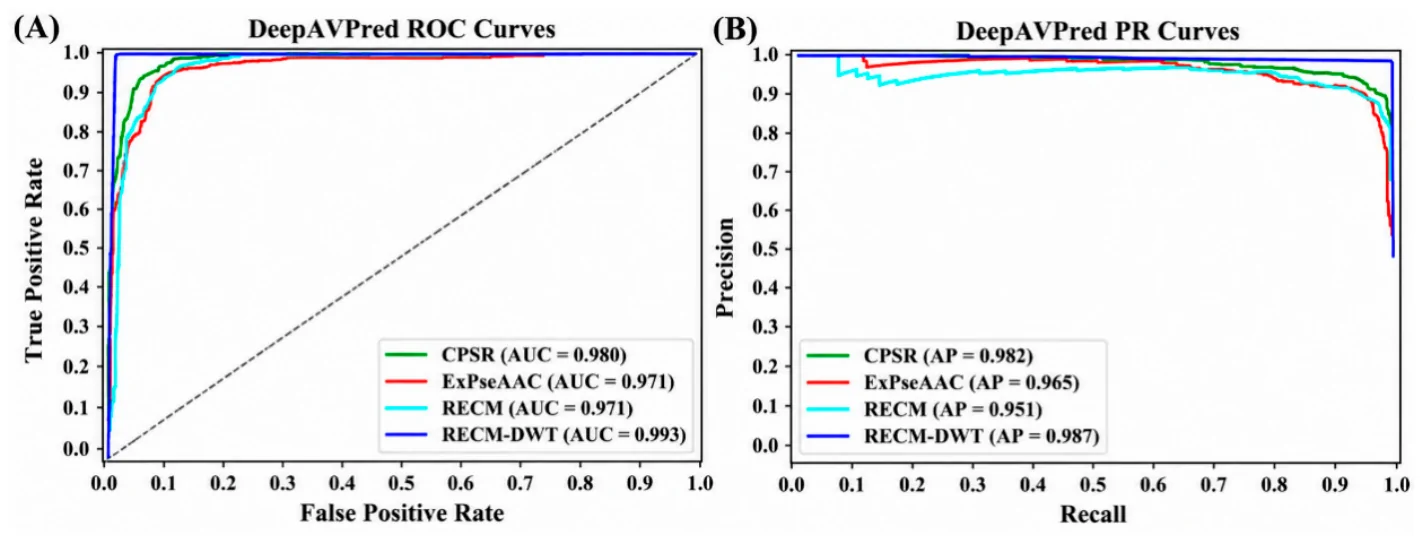
Receiver operating characteristic (ROC) and precision–recall (PR) curve analysis using different feature-encoding schemes. (**A**) ROC curves for CPSR, ExPseAAC, RECM, and RECM-DWT. (**B**) PR curves for CPSR, ExPseAAC, RECM, and RECM-DWT.

As shown in Figure 1A, all models demonstrate strong discriminative ability based on their ROC curves. Among the compared methods, RECM-DWT achieved the highest area under the ROC curve (AUC = 0.993), outperforming CPSR (AUC = 0.980), ExPseAAC (AUC = 0.971), and RECM (AUC = 0.971). The higher AUC value indicates that RECM-DWT better distinguished between positive and negative samples across different decision thresholds than the other evaluated feature representations. Notably, the steep initial ascent of the RECM-DWT curve reflects strong sensitivity even under stringent false-positive constraints. Figure 1B presents the Precision–Recall (PR) curves, which are particularly informative for imbalanced datasets commonly encountered in classification tasks. Consistent with the ROC analysis, RECM-DWT achieved the highest average precision (AP = 0.987), followed by CPSR (AP = 0.982), ExPseAAC (AP = 0.965), and RECM (AP = 0.951). The PR curve of RECM-DWT remained above those of the other feature representations across most recall levels, indicating better precision retention as sensitivity increases. 

### 2.5. Comparison with Existing State-of-the-Art AVP Predictors

To further assess the performance of DeepAVPred, its results were compared with previously reported results from existing AVP prediction methods, including AVPred, Chang et al.’s method [14], Meta-iAVP, DeepAVP, and ENNAVIA. The comparative results on AVP_train_ and AVP_test_ are summarized in Table 5. On the AVP_train_ dataset, DeepAVPred achieved an accuracy of 0.990 and an MCC of 0.980, while on the independent AVP_test_ dataset, it achieved an accuracy of 0.942 and an MCC of 0.886.

### 2.6. In Silico Allergenicity and Toxicity Assessment of Antiviral Peptides

For post-prediction characterization, 29 of the 60 positive AVP sequences in the AVP_test_ dataset, which received strong antiviral prediction outputs from DeepAVPred, were prioritized for subsequent analysis. These selected peptides were cross-referenced with the SATPdb database to examine previously reported antiviral activity and corresponding database records. The remaining 31 positive AVP_test_ sequences were not carried forward to further structural and in silico safety analyses. These peptides were subsequently subjected to structural modeling and in silico assessments of allergenicity and toxicity, with the complete results provided in Supplementary Tables S4 and S5. Among the 29 evaluated peptides, 18 were predicted to be non-allergenic and 25 were predicted to be non-toxic. When both criteria were considered simultaneously, 16 peptides were predicted to be both non-allergenic and non-toxic. Accordingly, these 16 peptides were retained as candidates for further investigation based on the combined in silico screening criteria.

## 3. Discussion

### 3.1. Interpretation of Feature-Representation Performance

The CPSR results suggest that CPSR features benefit from models capable of capturing nonlinear relationships. The independent-test results for CPSR indicate competitive performance with ensemble-based ML classifiers, while the deep models showed comparatively lower performance. Overall, CPSR provides a reasonable baseline feature representation; however, its performance varies depending on the learning algorithm.

The ExPseAAC results indicate that ExPseAAC provided physicochemical and sequence-order information that can be utilized within the evaluated classification framework. These findings indicate that ExPseAAC provided useful predictive performance on AVP_test_; however, the results should be interpreted cautiously given the observed sequence similarity between AVP_train_ and AVP_test_. Nevertheless, ExPseAAC showed lower overall performance than the energy-based feature representations in the present experiments.

The RECM results suggest that RECM features perform effectively with ensemble-based classifiers. However, although RECM improves predictive performance, it does not explicitly capture frequency-domain or multi-scale patterns, which may limit its discriminative power.

The performance gain achieved with RECM-DWT highlights the effectiveness of integrating frequency-domain information with residue interaction energies.

The precision–recall results suggest that RECM-DWT helps maintain a favorable precision–recall balance, which is important for prioritizing candidate AVPs for downstream validation.

### 3.2. Comparison with Existing AVP Predictors

The performance values of the existing AVP predictors were obtained from their respective published studies. The results reported here were generated with the same benchmark datasets, but differences in data preprocessing, partitioning of the datasets, model implementation, and evaluation procedures may impact comparability. Therefore, Table 5 provides a contextual comparison with previously reported results rather than a strictly controlled head-to-head comparison.

Overall, the results indicate that DeepAVPred achieves competitive predictive performance compared with previously reported AVP predictors.

### 3.3. Limitations and Future Validation

The RECM-DWT evaluations of LightGBM and MLP demonstrated a strong positive-class prediction bias on AVP_test_, particularly for LightGBM. The available results do not establish a specific underlying cause for this behavior; therefore, we avoid attributing the observed deterioration to a particular model- or feature-related mechanism.

The results in Section 2.4 demonstrate a performance improvement associated with the use of RECM-DWT features within the proposed Deep BiGRU framework. However, because the attention component was not independently ablated, its individual contribution to the observed predictive performance cannot be quantitatively determined from the present results. A component-level ablation of the attention mechanism would provide a more detailed assessment of its contribution in future work.

The cross-validation performance was satisfactory, but the existing experimentally validated AVP dataset remains relatively small and lacks sufficient diversity. The evaluation of model generalizability is strengthened by the inclusion of a balanced dataset of 1088 sequences (544 AVPs and 544 non-AVPs); however, larger and more diverse experimentally validated datasets would provide further validation of model generalizability.

However, several limitations of this study should be noted. First, the number of experimentally validated antiviral peptides available in public databases remains relatively small compared with the number of peptides or proteins available in these databases, which may limit the generalizability of the deep learning model. The limited size and diversity of the training dataset may increase uncertainty in performance estimates and restrict the model’s generalization to peptides that differ substantially from those represented in the training data. The current cross-validation results alone do not establish that the sample size is sufficient. To evaluate model performance, 10-fold cross-validation was performed on AVP_train_, while AVP_test_ was used separately for independent performance evaluation. To reduce sequence redundancy, CD-HIT with a sequence-identity cutoff of 0.5 was applied during dataset preparation. Additional CD-HIT-2D analysis showed that 67 of the 120 AVP_test_ sequences (55.8%) clustered with sequences in AVP_train_ at the 0.5 identity threshold, indicating substantial sequence similarity between the datasets. Therefore, the independent-test performance should be interpreted with this limitation in mind. As more antiviral peptides are experimentally validated and added to public databases, larger and more diverse datasets will support future evaluation.

### 3.4. Implications of In Silico Peptide Assessment

These computational predictions represent preliminary assessments and do not establish pharmacokinetic properties or in vivo safety. Experimental validation is required to confirm their allergenicity, toxicity, and suitability for further therapeutic investigation.

## 4. Materials and Methods

### 4.1. Data Collection and Preprocessing

The development of an effective computational predictor requires a reliable and well-curated benchmark dataset. In this study, we adopted the benchmark datasets previously used by Thakur et al. [13], facilitating comparison with previously reported results. Antiviral peptides (AVPs) are short peptide sequences composed of the 20 standard amino acids and are represented in FASTA format. The AVP_train_ and AVP_test_ subsets were adopted from the previously published AVPpred benchmark dataset [13]. Because the available records of the original benchmark preparation were insufficient to determine the extent of sequence similarity between the published training and validation/test subsets, we additionally assessed potential cross-set sequence overlap during the present revision. Specifically, AVP_train_ was used as the reference database and AVP_test_ as the query dataset for CD-HIT-2D analysis using a sequence identity threshold of 0.5. The results of this additional analysis are described below. The non-AVP sequences used in this study were adopted from the benchmark dataset reported in the AVPPred study. According to the original study, the non-AVP dataset was constructed from experimentally validated non-secretory peptides obtained from public protein databases [13]. To prevent overlap in activity types and to obtain a reliable set of negative samples for model training and evaluation, peptides with known therapeutic peptide function (other than antiviral) were omitted. The final dataset was divided into two subsets: AVP_train_ and AVP_test_. AVP_train_ consisted of 1088 sequences, including 544 AVPs and 544 non-AVPs, and was used for model training and cross-validation. AVP^+^_train_ represents the AVP subset, whereas AVP^−^_train_ represents the non-AVP subset, and ∪ denotes set union. This balanced composition reduces potential bias due to class imbalance.

(1) $${\mathrm{AVP}}_{\mathrm{train}}={\mathrm{AVP}}_{\mathrm{train}}^{+}\cup {\mathrm{AVP}}_{\mathrm{train}}^{-}$$

AVP_test_ corresponds to the published validation/test subset and contains 120 sequences, comprising 60 AVPs and 60 non-AVPs. In the present study, model training and 10-fold cross-validation were conducted using AVP_train_, whereas AVP_test_ was used for separate performance evaluation of the different feature representations and classification models reported in Table 1, Table 2, Table 3 and Table 4. The available records do not provide sufficient information to establish whether AVP_test_ results were consulted during historical feature or model selection, hyperparameter tuning, threshold setting, early stopping, or other model-development decisions. Therefore, AVP_test_ is not described as being used exclusively for final model evaluation or as completely independent of model-development decisions. ${\mathrm{AVP}}_{\mathrm{test}}^{+}$ denotes the AVPs as a positive class, whereas ${\mathrm{AVP}}_{\mathrm{test}}^{-}$ denotes the non-AVPs as a negative class in the AVP_test_ dataset.

(2) $${\mathrm{AVP}}_{\mathrm{test}}={\mathrm{AVP}}_{\mathrm{test}}^{+}\cup {\mathrm{AVP}}_{\mathrm{test}}^{-}$$

To further assess potential sequence overlap between AVP_train_ and AVP_test_, an additional CD-HIT-2D analysis was performed using AVP_train_ as the reference dataset and AVP_test_ as the query dataset at a sequence identity threshold of 0.5 (-c 0.5, -n 3). Of the 120 AVP_test_ sequences, 67 (55.8%) were clustered with sequences in AVP_train_ under the specified CD-HIT-2D criterion, whereas 53 (44.2%) were not clustered. These results indicate substantial sequence similarity between AVP_train_ and AVP_test_, and this limitation should therefore be considered when interpreting AVP_test_ performance. Although the current evaluation used the original 10-fold cross-validation protocol, similarity-aware or cluster-based cross-validation would provide a more stringent assessment of model generalization by reducing sequence similarity between training and validation subsets. This represents a limitation of the current evaluation and highlights an important direction for future validation.

### 4.2. Feature Extraction Strategies

Feature representation plays a critical role in the performance of machine learning and deep learning models [24]. To comprehensively characterize peptide sequences, we employed four complementary feature-encoding strategies: Extended Pseudo Amino Acid Composition (ExPseAAC), Composite Protein Sequence Representation (CPSR), Residue Energy Content Matrix (RECM), and a newly proposed RECM enhanced with Discrete Wavelet Transform (RECM-DWT). These descriptors were designed to capture diverse physicochemical, structural, and interaction-based properties of peptide sequences.

#### 4.2.1. Extended Pseudo Amino Acid Composition (ExPseAAC)

Pseudo-amino acid composition (PseAAC) extends conventional amino acid composition by incorporating sequence-order and physicochemical information. Based on the original PseAAC concept, we developed an extended version by integrating additional physicochemical properties, including rigidity, flexibility, hydrophilicity, hydrophobicity, and irreplaceability of amino acids. These properties play an important role in determining peptide structure and biological function [25,26,27,28]. As a result, each peptide sequence was represented by a 45-dimensional feature vector, calculated as (20 + 5 × 5), where 20 corresponds to the standard amino acid composition, and 5 × 5 represents sequence-order correlation features derived from five selected physicochemical properties over five tiers. Let P = (P_1_, P_2_, …, P_n_) denote a peptide sequence of length N, where P_i_ represents the i-th amino acid residue. The resulting ExPseAAC feature vector captures both amino acid composition and physicochemical correlations within the sequence. Specifically, the first 20 components represent the frequencies of the 20 standard amino acids and thus describe amino acid composition without preserving residue order. The remaining 25 components are sequence-order correlation factors derived from five physicochemical properties at five sequence lags. These factors summarize correlations between residues separated by a given sequence lag. Therefore, two peptides with the same amino acid composition but different residue orders can have different correlation values. The correlation factors reflect relative sequence-order information rather than the absolute positions of individual residues.

#### 4.2.2. Composite Protein Sequence Representation (CPSR)

Composite Protein Sequence Representation (CPSR) is a feature extraction method designed to encode diverse biochemical and structural characteristics of peptide sequences. CPSR consists of seven groups of attributes: amino acid composition, sequence length, 2-g exchange group frequency, hydrophobic group, electronic group, sum of hydrophobicity, and R-group composition [29,30]. This feature extraction strategy has been successfully applied in our previous studies for predicting various protein and peptide properties, including cell-penetrating peptides and defensin peptides. In this study, CPSR was employed to extract informative patterns from AVP sequences and to provide a comprehensive representation for classification tasks. Details of the physicochemical properties used for CPSR feature construction are provided in Supplementary Table S1. These groups are concatenated to give a 71-dimensional feature vector (20 + 1 + 36 + 2 + 6 + 1 + 5 = 71 attributes).

#### 4.2.3. Residue Energy Content Matrix (RECM)

Pairwise residue interaction energies provide valuable insights into peptide folding and structural stability [31]. The Residue Energy Content Matrix (RECM), originally proposed by Dosztányi et al. [32], was employed to capture residue–residue contact energy information derived from experimentally solved protein structures. RECM is a 20 × 20 matrix in which each row and column corresponds to one of the 20 standard amino acids, and each matrix element represents the estimated interaction energy between two residues. By mapping peptide sequences to RECM-based numerical representations, important energetic characteristics related to peptide structure and function can be effectively encoded [33]. The RECM property values of 20 amino acids used in this study are provided in Supplementary Table S2. For a peptide sequence of length *L*, let *n*_a_ denote the number of occurrences of amino-acid type *a*, and let *E_ab_* represent the interaction-energy value between amino-acid types *a* and *b* in the 20 × 20 residue-energy table. The sequence-specific RECM feature was calculated as

(3) $$R_{\mathrm{ab}}=\frac{n_{a}E_{\mathrm{ab}}}{L},\;a,b=1,\ldots ,20$$

Thus, each amino acid type occurring in the peptide contributes according to its frequency and the corresponding row of the residue-energy table. The resulting 20 × 20 representation was flattened by concatenating its rows to produce a fixed-length 400-dimensional feature vector. Division by *L* normalizes the representation with respect to peptide length, allowing peptides of different lengths to be represented with the same feature dimension.

For example, consider the peptide ACA, which has length *L* = 3. It contains two alanine (A) residues and one cysteine (C) residue. Therefore, *n*_A_ = 2, *n*_C_ = 1, and the counts of the remaining amino acid types are zero. Accordingly, the entries associated with alanine and cysteine are calculated as *R*_AB_ = 2*E_AB_/3* and R_Cb_ = E_Cb_/3, respectively, for *b* = 1…, 20, whereas rows corresponding to amino acid types absent from the peptide contain zero values. Consequently, every peptide, regardless of its original sequence length, is represented by the same 20 × 20 (400-dimensional) RECM feature representation.

#### 4.2.4. Discrete Wavelet Transform-Based RECM Feature (RECM-DWT)

**Figure 2:**
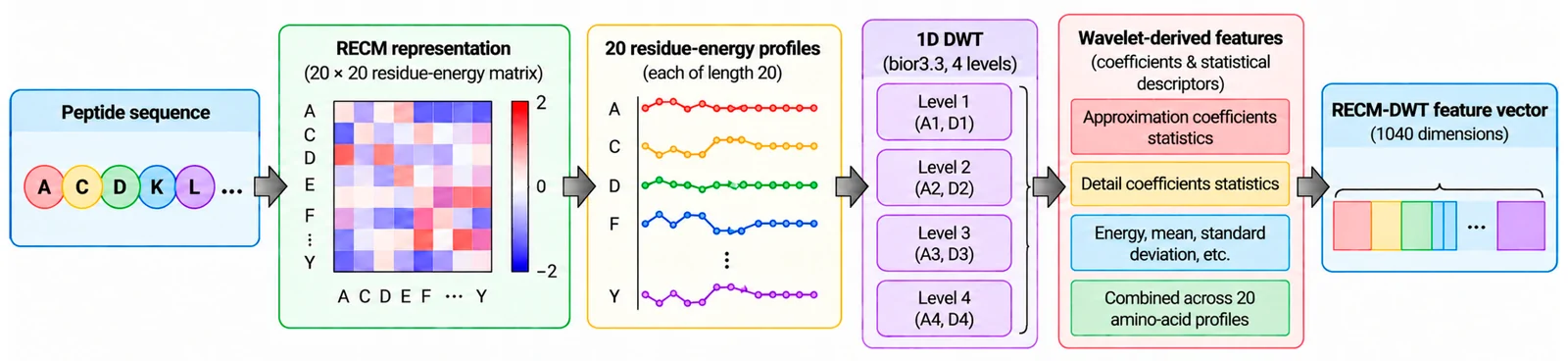
Schematic representation of the RECM-DWT feature-extraction procedure. The peptide sequence is first encoded as 20 residue-energy profiles using RECM. A one-dimensional discrete wavelet transform with the bior3.3 wavelet is then applied separately to each profile over four decomposition levels. Wavelet-derived coefficients and statistical descriptors from the 20 profiles are combined to produce a fixed 1040-dimensional RECM-DWT feature vector.

To further characterize multi-scale patterns in residue-energy information, a discrete wavelet transform (DWT)-based representation, termed RECM-DWT, was employed. DWT provides a multi-resolution analysis of numerical signals by decomposing them into approximation and detail components at successive resolution levels [34]. In the RECM-DWT feature-extraction procedure, a one-dimensional DWT using the biorthogonal bior3.3 wavelet was applied separately to each residue-energy profile over four successive decomposition levels. At each decomposition level, wavelet-derived coefficients and statistical descriptors were extracted from the residue-energy profiles and combined across the 20 amino acid profiles to obtain a fixed-dimensional representation. The overall RECM-DWT feature-extraction workflow is illustrated in Figure 2.

The procedure generated a 1040-dimensional RECM-DWT feature vector for each peptide, irrespective of the original peptide length. Consequently, peptides of different lengths were represented in a common fixed-dimensional feature space. The resulting RECM-DWT feature matrices had dimensions of 1088 × 1040 for AVP_train_ and 120 × 1040 for AVP_test_, corresponding to 1040 features per peptide.

### 4.3. Proposed Model Architecture

**Figure 3:**
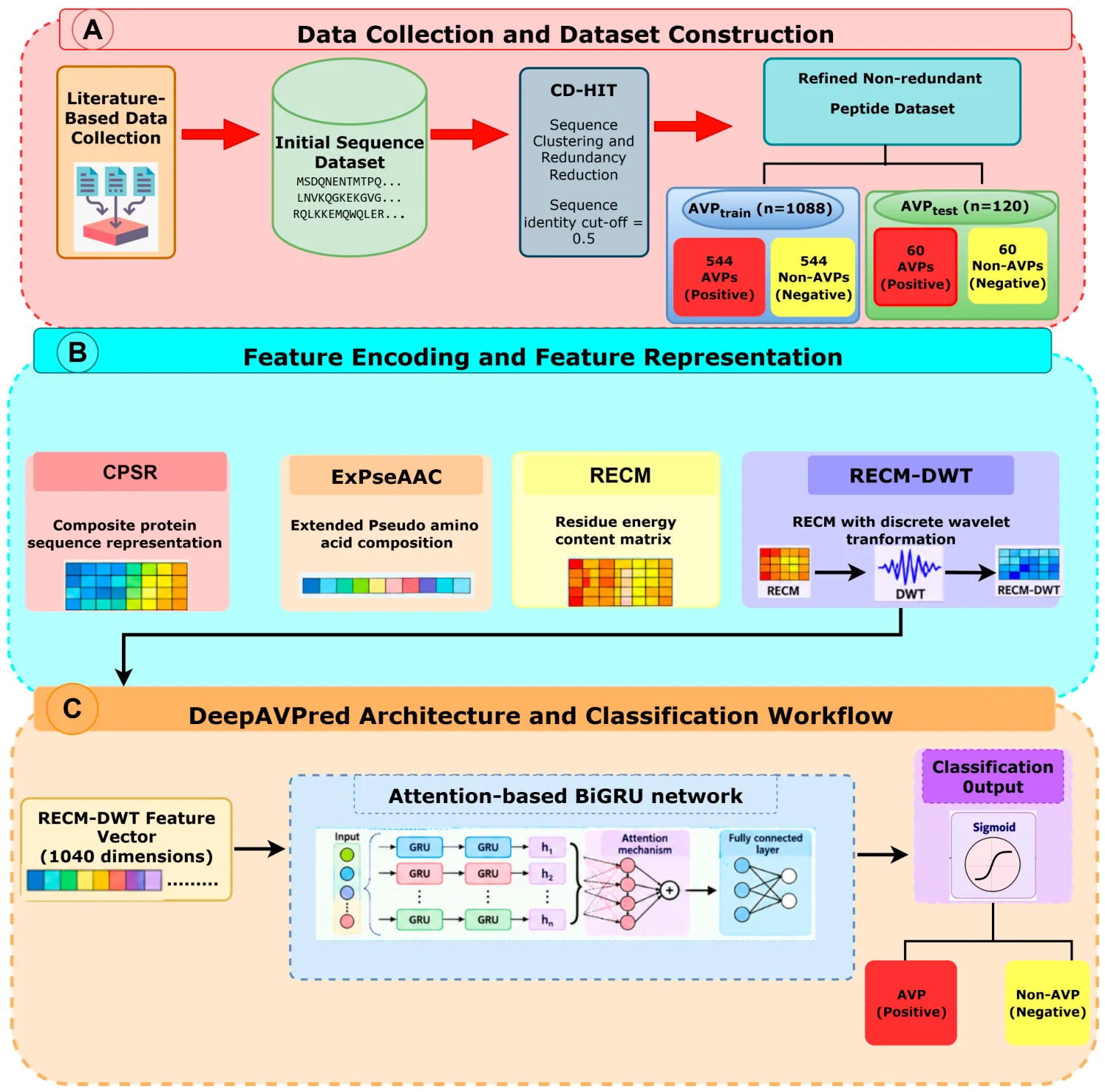
Overview of the DeepAVPred framework for antiviral peptide prediction. The workflow includes (**A**) data collection, preprocessing, and dataset information; (**B**) feature encoding; and (**C**) model architecture and classification workflow.

DeepAVPred is a deep learning framework based primarily on a Bidirectional Gated Recurrent Unit (BiGRU) architecture. For the RECM-DWT representation, the RECM-derived residue-energy information is organized into 20 one-dimensional residue-energy profiles. A one-dimensional discrete wavelet transform (DWT) using the biorthogonal bior3.3 wavelet is applied separately to each residue-energy profile over four successive decomposition levels. Wavelet-derived coefficients and statistical descriptors are then combined across the 20 profiles to generate a fixed 1040-dimensional RECM-DWT feature vector for each peptide. This fixed-dimensional representation is subsequently used for model training and classification. The proposed architecture employs BiGRU layers followed by an attention mechanism as components of the overall DeepAVPred framework for AVP classification. The final output layer uses a sigmoid activation function to classify peptide sequences as AVPs or non-AVPs. The complete DeepAVPred model workflow is illustrated in Figure 3, and the hyperparameters of the proposed model are provided in Supplementary Table S3.

### 4.4. Baseline Models for Comparison

To comprehensively evaluate the performance of DeepAVPred, several classical machine learning and deep learning models were implemented for comparison, including a Support Vector Machine (SVM), Random Forest (RF), Light Gradient Boosting Machine (LightGBM), a Multi-Layer Perceptron (MLP), and Deep Forest. All baseline models were trained and evaluated using the same datasets and feature representations to ensure a fair comparison.

### 4.5. Model Training and Evaluation

A 10-fold cross-validation strategy was employed on the AVP_train_ dataset to assess model stability and robustness. Performance was evaluated using commonly adopted metrics, including sensitivity (Sn), specificity (Sp), accuracy (Acc), and Matthews correlation coefficient (MCC). In addition, receiver operating characteristic (ROC) curve analysis and area under the curve (AUC) values were used to assess the discriminative capability of the models. The evaluation metrics were calculated as follows:

(4) $$\left\{\begin{array}{c} \mathrm{Sn}=\frac{\mathrm{TP}}{\mathrm{TP}+\mathrm{FN}}\\ \mathrm{Sp}=\frac{\mathrm{TN}}{\mathrm{TN}+\mathrm{FP}}\\ \mathrm{Acc}=\frac{\mathrm{TP}+\mathrm{TN}}{\mathrm{TP}+\mathrm{TN}+\mathrm{FP}+\mathrm{FN}}\\ \mathrm{MCC}=\frac{\mathrm{TP}\times \mathrm{TN}-\mathrm{FP}\times \mathrm{FN}}{\sqrt{\left(\mathrm{TP}+\mathrm{FN})\times (\mathrm{TN}+\mathrm{FN})\times (\mathrm{TP}+\mathrm{FP})\times (\mathrm{TN}+\mathrm{FP}\right)}} \end{array}\right.$$

where TP, FN, TN, and FP represent the number of true positives, false negatives, true negatives, and false positives, respectively. Sensitivity (Sn) and specificity (Sp) reflect the ability of the model to accurately identify AVPs and non-AVPs, respectively. Accuracy (Acc) indicates the model’s overall ability to correctly distinguish between AVP and non-AVP sequences. The Matthews correlation coefficient (MCC) is a widely used metric in machine learning to assess the performance of classification models. Furthermore, receiver operating characteristic (ROC) curve analysis provides an effective approach for comparing different models. The ROC curve provides a visual representation of the classification performance and discriminative capability of predictive models.

### 4.6. Structural Modeling and In Silico Allergenicity and Toxicity Assessment of Antiviral Peptides

For post-prediction characterization, 29 of the 60 positive AVP sequences in the AVP_test_ dataset, which received strong antiviral prediction from DeepAVPred, were prioritized for subsequent analysis. These selected peptides were cross-referenced with the SATPdb database to examine previously reported antiviral activity and corresponding database records. The remaining 31 positive AVP_test_ sequences were not carried forward to further structural and in silico safety analyses. These peptides were subsequently subjected to structural modeling and in silico assessments of allergenicity and toxicity. The three-dimensional structures of the 29 peptides were predicted using AlphaFold and subsequently refined through geometry optimization and energy minimization using PyMOL (version 1.7.4.5). The allergenicity of the peptides was assessed using AllerTOP v2.0, AllergenFP v1.0, and AlgPred v2.0, while toxicity was predicted using the ToxinPred web server with default parameters. These computational analyses represent preliminary in silico assessments of allergenicity and toxicity and do not establish the pharmacokinetic properties or in vivo safety of these peptides; experimental validation is required.

## 5. Conclusions

In this study, we proposed DeepAVPred, a deep learning-based computational framework for the accurate identification of antiviral peptides. DeepAVPred integrates advanced feature engineering with a Bidirectional Gated Recurrent Unit (BiGRU) architecture incorporating an attention mechanism for AVP classification. A key contribution of this work is the introduction of a novel feature representation, RECM enhanced with a Discrete Wavelet Transform (RECM-DWT), which incorporates multi-scale frequency-domain information from residue energy interactions. Comparative experiments showed that the RECM-DWT representation achieved strong predictive performance within the overall DeepAVPred framework compared with the other evaluated feature representations. Using RECM-DWT with the proposed DeepAVPred architecture, the framework achieved an accuracy of 0.990 in 10-fold cross-validation and 0.942 on the AVP_test_ dataset. Comparison with previously reported results from existing AVP predictors indicates that DeepAVPred achieved competitive predictive performance; however, differences in preprocessing and evaluation protocols should be considered when interpreting these comparisons. Because the attention component was not independently ablated, its individual contribution to the observed predictive performance cannot be quantitatively determined from the present results. A component-level ablation of the attention mechanism would provide a more detailed assessment of its contribution in future work. In addition to predictive performance evaluation, post-prediction characterization was performed through 3D structural modeling and in silico assessment of allergenicity and toxicity of experimentally validated antiviral peptides. Computational tools predicted most of the evaluated peptides to be non-allergenic and non-toxic. However, these results represent preliminary in silico assessments and do not establish pharmacokinetic properties or safety; experimental validation is required. Overall, DeepAVPred demonstrates promising performance as a computational framework for antiviral peptide identification and may serve as a useful tool to support peptide-based antiviral research. The results suggest its potential to capture relevant sequence and structural patterns associated with antiviral activity. Future work will focus on expanding the training dataset, incorporating additional structural and evolutionary features, and extending the framework to predict peptide activity against specific viral families and emerging mutant strains.

## Figures and Tables

**Table 1:** Performance of classification models using CPSR features on AVP_train_ (*n* = 1088) and AVP_test_ (*n* = 120) datasets.

Datasets	Models	Acc	Sn	Sp	MCC	AUC
AVP_train_	SVM	0.829	0.685	0.972	0.687	0.910
	MLP	0.870	0.878	0.862	0.741	0.936
	RF	0.894	0.863	0.924	0.791	0.957
	LightGBM	0.872	0.869	0.875	0.745	0.946
	Deep Forest	0.843	0.843	0.843	0.690	0.925
	Deep BiGRU	0.939 ± 0.040	0.925 ± 0.041	0.954 ± 0.045	0.880 ± 0.080	0.980 ± 0.029
AVP_test_	SVM	0.850	0.700	1.000	0.733	0.924
	MLP	0.891	0.866	0.916	0.784	0.958
	RF	0.933	0.866	1.000	0.874	0.962
	LightGBM	0.925	0.916	0.933	0.850	0.959
	Deep Forest	0.850	0.850	0.850	0.700	0.936
	Deep BiGRU	0.900	0.866	0.933	0.801	0.960

Note: Deep BiGRU’s performance on AVP_train_ is shown as mean ± SD over the 10 cross-validation folds.

**Table 2:** Performance of classification models using ExPseAAC features on AVP_train_ (*n* = 1088) and AVP_test_ (*n* = 120) datasets.

Datasets	Models	Acc	Sn	Sp	MCC	AUC
AVP_train_	SVM	0.791	0.769	0.812	0.584	0.883
	MLP	0.846	0.858	0.834	0.695	0.918
	RF	0.868	0.839	0.897	0.739	0.940
	LightGBM	0.863	0.872	0.852	0.727	0.938
	Deep Forest	0.834	0.823	0.845	0.670	0.902
	Deep BiGRU	0.921 ± 0.031	0.881 ± 0.031	0.961 ± 0.039	0.845 ± 0.062	0.971 ± 0.020
AVP_test_	SVM	0.791	0.769	0.812	0.584	0.883
	MLP	0.850	0.783	0.916	0.706	0.923
	RF	0.875	0.866	0.916	0.784	0.958
	LightGBM	0.900	0.833	0.966	0.800	0.952
	Deep Forest	0.833	0.750	0.916	0.676	0.898
	Deep BiGRU	0.858	0.833	0.883	0.717	0.903

Note: Deep BiGRU’s performance on AVP_train_ is shown as mean ± SD over the 10 cross-validation folds.

**Table 3:** Performance of classification models using RECM features on AVP_train_ (*n* = 1088) and AVP_test_ (*n* = 120) datasets.

Datasets	Models	Acc	Sn	Sp	MCC	AUC
AVP_train_	SVM	0.835	0.819	0.851	0.671	0.905
	MLP	0.858	0.843	0.873	0.717	0.916
	RF	0.868	0.858	0.878	0.739	0.947
	LightGBM	0.859	0.858	0.860	0.720	0.937
	Deep Forest	0.833	0.782	0.884	0.672	0.916
	Deep BiGRU	0.921 ± 0.029	0.907 ± 0.056	0.936 ± 0.023	0.844 ± 0.056	0.971 ± 0.023
AVP_test_	SVM	0.841	0.800	0.883	0.685	0.890
	MLP	0.883	0.816	0.950	0.773	0.936
	RF	0.900	0.833	0.966	0.807	0.936
	LightGBM	0.883	0.816	0.950	0.773	0.933
	Deep Forest	0.833	0.716	0.950	0.685	0.892
	Deep BiGRU	0.850	0.816	0.883	0.701	0.896

Note: Deep BiGRU’s performance on AVP_train_ is shown as mean ± SD over the 10 cross-validation folds.

**Table 4:** Performance of classification models using RECM-DWT features on AVP_train_ (*n* = 1088) and AVP_test_ (*n* = 120) datasets.

Datasets	Models	Acc	Sn	Sp	MCC	AUC
AVP_train_	SVM	0.912	0.902	0.922	0.832	0.979
	MLP	0.928	0.917	0.939	0.857	0.971
	RF	0.908	0.907	0.908	0.826	0.979
	LightGBM	0.954	0.909	1.000	0.912	1.000
	Deep Forest	0.912	0.896	0.928	0.832	0.975
	Deep BiGRU	0.990 ± 0.032	0.987 ± 0.040	0.993 ± 0.023	0.980 ± 0.063	0.993 ± 0.021
AVP_test_	SVM	0.933	0.900	0.966	0.868	0.994
	MLP	0.608	0.950	0.266	0.296	0.797
	RF	0.916	0.883	0.950	0.835	0.970
	LightGBM	0.500	1.000	0.000	0.000	0.604
	Deep Forest	0.916	0.950	0.883	0.835	0.980
	Deep BiGRU	0.942	0.983	0.900	0.886	0.949

Note: Deep BiGRU’s performance on AVP_train_ is shown as mean ± SD over the 10 cross-validation folds.

**Table 5:** Contextual comparison of DeepAVPred with previously reported results of existing AVP predictors on AVP_train_ (*n* = 1088) and AVP_test_ (*n* = 120) datasets.

Dataset	Predictor	Model	Acc	Sn	Sp	MCC
AVP_train_	AVPred	SVM	0.900	0.900	0.903	0.800
	Chang et al. [14]	RF	0.915	0.915	0.941	0.830
	Meta-iAVP	Meta-predictor	0.932	0.932	0.974	0.870
	DeepAVP	Dual-channel	0.901	0.901	0.908	0.880
	ENNAVIA	DNN	0.959	0.959	0.984	0.920
	DeepAVPred	Deep BiGRU	0.990	0.987	0.993	0.980
AVP_test_	AVPred	SVM	0.925	0.933	0.917	0.850
	Chang et al. [14]	RF	0.930	0.917	0.950	0.870
	Meta-iAVP	Meta-predictor	0.949	0.917	0.983	0.900
	DeepAVP	Dual-channel	0.933	0.967	0.900	0.870
	ENNAVIA	DNN	0.938	0.947	0.927	0.870
	DeepAVPred	Deep BiGRU	0.942	0.983	0.900	0.886

## Data Availability

The code and scripts supporting this study are publicly available on GitHub at: https://github.com/Huma-fida01/DeepAVPred.

## Supplementary Materials

The following supporting information can be downloaded at: https://www.mdpi.com/article/10.3390/ijms27209086/s1.

## Abbreviations

The following abbreviations are used in this manuscript:

Acc	accuracy
AIDS	acquired immunodeficiency syndrome
AP	average precision
AUC	area under the receiver operating characteristic curve
AVP	antiviral peptide
AVP_test_	independent test dataset for antiviral peptide prediction
AVP_train_	training dataset for antiviral peptide prediction
BiGRU	bidirectional gated recurrent unit
CD-HIT	Cluster Database at High Identity with Tolerance
COVID-19	coronavirus disease 2019
CPSR	composite protein sequence representation
DL	deep learning
DNN	deep neural network
DWT	discrete wavelet transform
ExPseAAC	extended pseudo amino acid composition
FASTA	FASTA sequence format
FN	false negative
FP	false positive
HH	diagonal high-frequency detail component
HIV	human immunodeficiency virus
HL	vertical high-frequency detail component
LH	horizontal high-frequency detail component
LightGBM	light gradient boosting machine
LL	low-frequency approximation component
MCC	Matthews correlation coefficient
ML	machine learning
MLP	multi-layer perceptron
non-AVP	non-antiviral peptide
PR	precision–recall
PseAAC	pseudo amino acid composition
RECM	residue energy content matrix
RECM-DWT	residue energy content matrix augmented with discrete wavelet transform
RF	random forest
ROC	receiver operating characteristic
Sn	sensitivity
Sp	specificity
SVM	support vector machine
TN	true negative
TP	true positive
