# Supplementary material for: DeepAVPred: A Deep Learning Framework Integrating RECM-DWT and BiGRU for Accurate Antiviral Peptide Prediction
Source: Int J Mol Sci. 2026 Oct 13;27(20):9086. doi: 10.3390/ijms27209086 (PMC13704966; doi:10.3390/ijms27209086)
Supplement: Supplementary file 1 [file ijms-27-09086-s001.zip › ijms-4553359-supplementary.pdf]

Supplementary Material

Table S1. Composite Protein Sequence Representation (CPSR) Feature Properties. The seven groups of attributes and their dimensions are listed; the final CPSR feature vector is concatenating all seven groups yielding a 71-dimensional vector (20+1+36+2+6+1+5=71 attributes).

Feature set	Number of attributes
Amino acid composition	20
Sequence length	1
2-Gram exchange group frequency	36
Hydrophobic group	2
Electronic group	6
Sum of hydrophobicity	1
R-group	5

Table S2. RECM property values of the twenty amino acids used in this study.

	**A**	**C**	**D**	**E**	**F**	**G**	**H**	**I**	**K**	**L**	**M**	**N**	**P**	**Q**	**R**	**S**	**T**	**V**	**W**	**Y**
**A**	-1.65	-2.83	1.16	1.8	-3.73	-0.41	1.9	-3.69	0.49	-3.01	-2.08	0.66	1.54	1.2	0.98	-0.08	0.46	-2.31	0.32	-4.62
**C**	-2.83	-39.58	-0.82	-0.53	-3.07	-2.96	-4.98	0.34	-1.38	-2.15	1.43	-4.18	-2.13	-2.91	-0.41	-2.33	-1.84	-0.16	4.26	-4.46
**D**	1.16	-0.82	0.84	1.97	-0.92	0.88	-1.07	0.68	-1.93	0.23	0.61	0.32	3.31	2.67	-2.02	0.91	-0.65	0.94	-0.71	0.90
**E**	1.8	-0.53	1.97	1.45	0.94	1.31	0.61	1.3	-2.51	1.14	2.53	0.2	1.44	0.1	-3.13	0.81	1.54	0.12	-1.07	1.29
**F**	-3.73	-3.07	-0.92	0.94	-11.25	0.35	-3.57	-5.88	-0.82	-8.59	-5.34	0.73	0.32	0.77	-0.4	-2.22	0.11	-7.05	-7.09	-8.80
**G**	-0.41	-2.96	0.88	1.31	0.35	-0.2	1.09	-0.65	-0.16	-0.55	-0.52	-0.32	2.25	1.11	0.84	0.71	0.59	-0.38	1.69	-1.90
**H**	1.9	-4.98	-1.07	0.61	-3.57	1.09	1.97	-0.71	2.89	-0.86	-0.75	1.84	0.35	2.64	2.05	0.82	-0.01	0.27	-7.58	-3.20
**I**	-3.69	0.34	0.68	1.3	-5.88	-0.65	-0.71	-6.74	-0.01	-9.01	-3.62	-0.07	0.12	-0.18	0.19	-0.15	0.63	-6.54	-3.78	-5.26
**K**	0.49	-1.38	-1.93	-2.51	-0.82	-0.16	2.89	-0.01	1.24	0.49	1.61	1.12	0.51	0.43	2.34	0.19	-1.11	0.19	0.02	-1.19
**L**	-3.01	-2.15	0.23	1.14	-8.59	-0.55	-0.86	-9.01	0.49	-6.37	-2.88	0.97	1.81	-0.58	-0.6	-0.41	0.72	-5.43	-8.31	-4.90
**M**	-2.08	1.43	0.61	2.53	-5.34	-0.52	-0.75	-3.62	1.61	-2.88	-6.49	0.21	0.75	1.9	2.09	1.39	0.63	-2.59	-6.88	-9.73
**N**	0.66	-4.18	0.32	0.2	0.73	-0.32	1.84	-0.07	1.12	0.97	0.21	0.61	1.15	1.28	1.08	0.29	0.46	0.93	-0.74	0.93
**P**	1.54	-2.13	3.31	1.44	0.32	2.25	0.35	0.12	0.51	1.81	0.75	1.15	-0.42	2.97	1.06	1.12	1.65	0.38	-2.06	-2.09
**Q**	1.2	-2.91	2.67	0.1	0.77	1.11	2.64	-0.18	0.43	-0.58	1.9	1.28	2.97	-1.54	0.91	0.85	-0.07	-1.91	-0.76	0.01
**R**	0.98	-0.41	-2.02	-3.13	-0.4	0.84	2.05	0.19	2.34	-0.6	2.09	1.08	1.06	0.91	0.21	0.95	0.98	0.08	-5.89	0.36
**S**	-0.08	-2.33	0.91	0.81	-2.22	0.71	0.82	-0.15	0.19	-0.41	1.39	0.29	1.12	0.85	0.95	-0.48	-0.06	0.13	-3.03	-0.82
**T**	0.46	-1.84	-0.65	1.54	0.11	0.59	-0.01	0.63	-1.11	0.72	0.63	0.46	1.65	-0.07	0.98	-0.06	-0.96	1.14	-0.65	-0.37
**V**	-2.31	-0.16	0.94	0.12	-7.05	-0.38	0.27	-6.54	0.19	-5.43	-2.59	0.93	0.38	-1.91	0.08	0.13	1.14	-4.82	-2.13	-3.59
**W**	0.32	4.26	-0.71	-1.07	-7.09	1.69	-7.58	-3.78	0.02	-8.31	-6.88	-0.74	-2.06	-0.76	-5.89	-3.03	-0.65	-2.13	-1.73	-12.39
**Y**	-4.62	-4.46	0.9	1.29	-8.8	-1.9	-3.2	-5.26	-1.19	-4.9	-9.73	0.93	-2.09	0.01	0.36	-0.82	-0.37	-3.59	-12.39	-2.68

Table S3. Parameter settings and libraries for BiGRU models using various features

Algorithms	Layers	Parameters	Libraries/package
BiGRU	Bi-GRU (64–256) → Attention → Bi-GRU (32–128) → Dense (1, sigmoid)	Adam (lr=1e-5–1e-2), L1/L2 (λ=0.001), dropout=0.2–0.5, batch=32, epochs=200, patience=5, Keras Tuner, loss = binary cross-entropy	TensorFlow/Keras, **Keras Tuner**, scikit-learn, NumPy, pandas, Matplotlib

Table S4. Three-Dimensional Structures of Experimentally Validated Antiviral Peptides from the SATPdb Database

Accession IDs	Peptides Sequence	3D Structure (Alpha Fold predications)
Satpdb13762	KIALLSTNKAVVSLSNGVSVLTSKVLDLKNYID	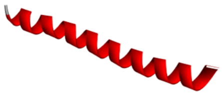
satpdb19910	YTSLIHSLIEESQNLQEKNEQELLELDKWASLWNWF	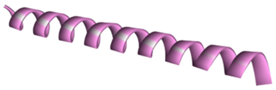
satpdb17662	KIPCGESCVWIPCLTSVFNCKCENKVCYHD	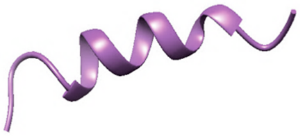
satpdb22382	YTSLIQSLIEESQNQQEKNEQQLLELDKWASLWNWF	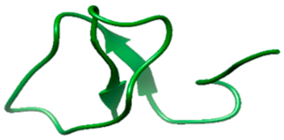
satpdb12980	GRFKRFRKPFKKLFKKIS	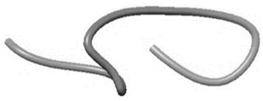
satpdb16491	LHRIDLGPPISLERLDVGTNLGNAIAKLEDAKELL	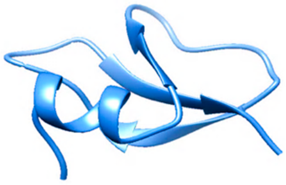

satpdb26982	VANDPIDISIELNKAKSDLEESKEWIRRSN QKLDSD	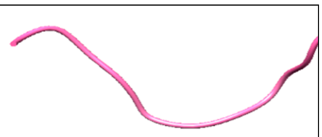
satpdb24088	RGGRLCYCRGWICFCVGR	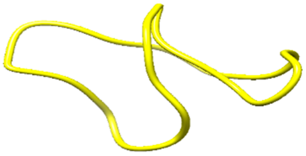
satpdb22686	FFLLFLQGAAGNSVLCRIRGGRCHVGSCHFPERHIGRCSGFQ ACCIRTWG	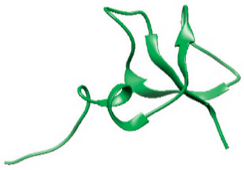
satpdb13590	TWLRAIWDWVCTALTDFK	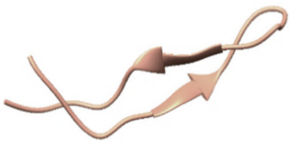
satpdb12291	LLGDLLRKSKEKIGKEFKRIVQRIKDFLRN LVPRTES	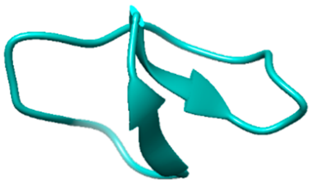
satpdb28004	WDREINNYTSLIHSLIEESQNQQEKNEQELLELDKW	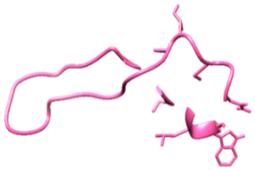
satpdb22712	AAHLIDALYAEFLGGRVLTTPVVHRALFYASAVLRQPFLAG VPSA	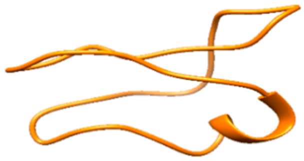
satpdb22618	KQTENLADTY	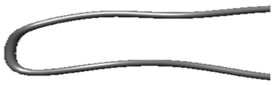

satpdb222<br>29	GICRCYCGRGICRCICGR	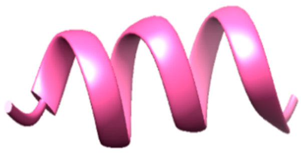
satpdb1513<br>0	SLSRFLSFLKIVYPPAF	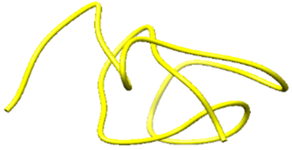
satpdb213<br>95	ATFNFINNCPFTVWAAAVPG	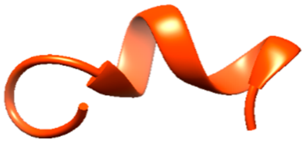
satpdb16088	WEYVLLLFLL	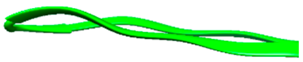
satpdb201<br>29	KQARSDIEKLKEAIRDTNKAVQSVQSSIGN<br>LIVAI	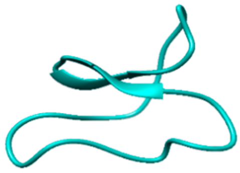
satpdb16492	VALDPIDISIELNKAKSDLEESKEWIRRSN<br>QKLDSD	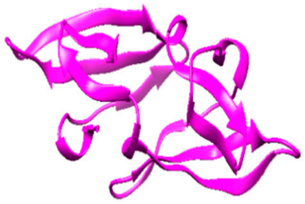
satpdb22193	CDVIALLACHLNT	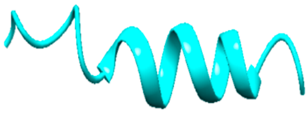
satpdb206<br>90	IWNHGNITLGEWYNQTKDLQQKFYEIITMD<br>IEQNNV	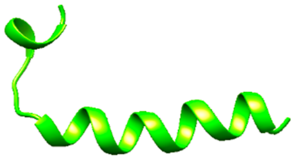

satpdb15297	RRKKAAVALLPAVLAALLAP	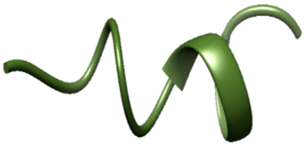
satpdb25293	**DCYCRIPACIAGERRYGTCIYQGRLWAFCC**	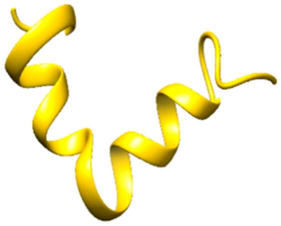
satpdb14659	TAAVALVEAKQARSDIEKLKEAIRDTNKAVQSVQS	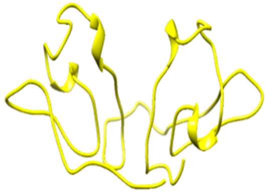
satpdb24699	YQLLIAMIYKNI	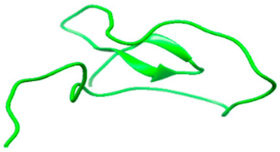
satpdb20302	PPISLERLDVGTNLGNAIAKLEDAKELLES SDQIL	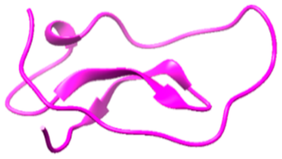
satpdb288 22	NYTSLIHSLIEESQNQQEKNEQELLELDKW ASLWNW	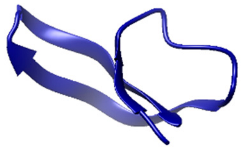
satpdb125 23	SWLRDLWDWICELLSDFK	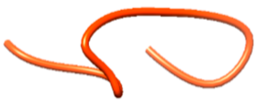

Table S5. Pharmacokinetic properties of the important antiviral peptides

**Peptide ID**	**Allergenicity**	**Hydrophobicity**	**Toxicity**	**Amphipathicity**	**Hydropathicity**	**Net Hydrogen**	**Molecular Weight**
Satpdb13762	Non-Allergen	-0.04	Non-Toxin	0.44	-0.18	0.73	3504.64
satpdb19910	Non-Allergen	-0.14	Non-Toxin	0.56	-0.09	0.92	4436.49
satpdb17662	Non-Allergen	-0.09	Non-Toxin	0.50	-0.20	0.63	3420.46
satpdb22382	Non-Allergen	-0.19	Non-Toxin	0.59	-0.88	1.03	4441.47
satpdb12980	Non-Allergen	-0.46	Non-Toxin	1.63	0.76	1.39	2312.15
satpdb16491	Allergen	-0.11	Non-Toxin	0.50	-0.01	0.71	3795.95
satpdb26982	*Non-Allergen*	0.18	Non-Toxin	0.26	-0.44	0.29	1493.00
satpdb24088	Non-Allergen	-0.19	Toxin	0.54	-0.29	1.00	2105.80
satpdb22686	Allergen	-0.09	Toxin	0.46	0.24	0.78	5527.22
satpdb13590	Allergen	-0.01	Non-Toxin	0.34	-0.60	0.78	2225.85

satpdb12291	Allergen	-0.03	Non-Toxin	0.37	0.58	0.60	3205.29
satpdb28004	Non-Allergen	-0.30	Non-Toxin	0.70	-1.35	1.14	4474.41
satpdb22712	Non-Allergen	0.06	Non-Toxin	0.28	0.77	0.53	4781.27
satpdb22618	Non-Allergen	-0.33	Non-Toxin	0.62	-1.50	1.1	1182.39
satpdb22229	Allergen	-0.22	Non-Toxin	0.54	0.42	0.94	1992.71
satpdb15130	Allergen	-0.40	Non-Toxin	2.10	-0.60	1.14	1722.60
satpdb21395	Allergen	0.14	Non-Toxin	0	0.73	0.45	2140.72
satpdb16088	Non-Allergen	0.36	Non-Toxin	0.13	2.03	0.3	1308.79
satpdb20129	Non-Allergen	-0.30	Non-Toxin	0.72	-0.89	1.03	4157.15
satpdb16492	Non-Allergen	0.04	Non-Toxin	0.3	0.64	0.55	5186.00
satpdb22193	Allergen	0.12	Non-Toxin	0.11	1.37	0.38	1385.85
satpdb20690	Allergen	-0.01	Non-Toxin	0.34	0.34	0.78	2225.85
satpdb15297	Non-Allergen	-0.02	Non-Toxin	0.61	0.61	0.6	2042.87

satpdb25293	Non-Allergen	-0.14	Toxin	0.41	0.41	0.83	3492.52
satpdb14659	Non-Allergen	-0.25	Non-Toxin	0.78	-0.4	0.97	3769.78
satpdb24699	Allergen	-0.11	Non-Toxin	0.62	-0.52	0.80	2274.91
satpdb20302	Allergen	-0.12	Toxin	0.50	-0.13	0.70	3763.80
satpdb28822	Non-Allergen	-0.19	Non-Toxin	0.72	-0.26	1.08	4556.97
satpdb12523	Non-Allergen	0.26	Non-Toxin	0.12	1.77	0.30	1026.46
